# Analytical Approaches for Deriving Friction Coefficients for Selected α-Helical Peptides Based Entirely on Molecular Dynamics Simulations

**DOI:** 10.1021/acs.jpcb.2c03076

**Published:** 2022-10-27

**Authors:** Aleksandra Wosztyl, Krzysztof Kuczera, Robert Szoszkiewicz

**Affiliations:** † Faculty of Chemistry, Biological and Chemical Research Centre, University of Warsaw, Żwirki i Wigury 101, 02-089Warsaw, Poland; ‡ Department of Chemistry, The University of Kansas, Lawrence, Kansas66045, United States; § Department of Molecular Biosciences, The University of Kansas, Lawrence, Kansas66045, United States

## Abstract

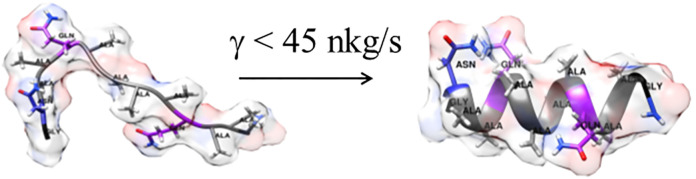

In this paper we derive analytically from molecular dynamics (MD) simulations the friction coefficients related to conformational transitions within several model peptides with α-helical structures. We study a series of alanine peptides with various length from ALA_5_ to ALA_21_ as well as their two derivatives, the (AAQAA)_3_ peptide and a 13-residue KR1 peptide that is a derivative of the (AAQAA)_2_ peptide with the formula GN(AAQAA)_2_G. We use two kinds of approaches to derive their friction coefficients. In the local approach, friction associated with fluctuations of single hydrogen bonds are studied. In the second approach, friction coefficients associated with a folding transitions within the studied peptides are obtained. In both cases, the respective friction coefficients differentiated very well the subtle structural changes between studied peptides and compared favorably to experimentally available data.

## Introduction

Helices are structural building blocks in many proteins and peptides, and the details of their folding are of fundamental interest to understand their function.^1^ This becomes particularly interesting in light of the recently renewed drive to understand the folding of short peptides under various conditions from the perspective of using them as modifiers for the adhesive and mechanical properties of arbitrary surfaces and various kinds of cells to be used in novel cancer therapies.^2,3^ Despite stunning computational progress, experimental approaches to study the folding of helical peptides are still lacking.^4^ This is mostly because helix folding and helical propagation at the single molecule level occur at the time scales of at most of tens of nanoseconds, which is too fast to be probed by the majority of experimental techniques.^4^ Therefore, alternative approaches and proxies to study helix folding are welcomed. In particular, since structural and mechanical properties are deeply connected, it makes sense to experimentally infer about folding from the related changes of selected mechanical properties during the folding process. Changes of the mechanical signature recently described by Ploscariu et al. would offer an interesting experimental approach to folding, since they dwell on the well-addressed viscoelastic Kelvin–Voigt model, where a protein is abstracted via dissipative springs, i.e., stiffnesses and their corresponding mechanical energy damping constants.^5^

At the heart of simplified mechanical signatures for peptides are estimates of the mechanical energy damping constants along a generalized folding coordinate.^5^ Such estimates relate to the molecular friction coefficient under particular experimental conditions. So far, there have been already some studies of the frictional coefficient for single peptides and proteins from both the experimental and theoretical/simulation perspectives.^6−15^ One type of approach dwells on models with dampened or driven harmonic oscillator with damping and yields the viscoelastic frictional (damping) coefficient in kg/s.^8,10,11,16,17^ Another type of approach yields various reconfiguration times for unfolded peptides and proteins in solution as well as folding/unfolding rate constants as a function of solvent viscosity.^7,12−14,18^ Relevant calculations of frictional coefficients, via various methods, dwell on solving various incarnations of Langevin equations.^19^ These are either Langevin equation for a dampened harmonic oscillator or Rouse-type models with internal friction.^10,20^ The first type of approach produces in equilibrium Einstein–Stokes fluctuation–dissipation relationships out of which relevant frictional coefficients are calculated from diffusion constants. The second type of model produces a spectrum of available reconfiguration times for various transition modes within the protein and predicts the linear dependence of reconfiguration modes on solvent viscosity in the case on unfolded proteins. Therein, internal friction of the molecule is also defined as reconfiguration times at the limit of vanishing solvent viscosity.

So far mostly linear, although sometimes exponential, relationships on solvent viscosity of either folding times or various relaxation/reconfiguration times have been observed both experimentally and via simulations for small peptides such as Trp cage, Ala_8_, (GlySer)_4_, and Ala_5_ as well as small proteins such as cold shock protein.^12,21,22^ In relation to helical peptides, typically observed values of internal friction were in the sub-nanosecond regime, while reconfiguration times of several nanoseconds were found at standard water viscosities. Furthermore, the available literature has identified that dihedral angle dynamics in peptides is one of the main causes for their friction and showed that such dynamics is strongly correlated with making or breaking of hydrogen bonds (however, mostly with the solvent).^12,23^

This work constitutes the first step toward developing a theoretical and experimental approach to fast folding α-helical peptides based on tracing their related changes of mechanical signatures during (un)folding reactions. Herein, friction coefficients, encompassing both the “solvent” and “internal” friction of the given peptide, are derived from molecular simulation results under typical simulation conditions for a series of alanine-based peptides. The novelty is to present two approaches uniquely combining existing models and use the parameters, which are able to relate such friction coefficients to single-molecule force spectroscopy experiments. We study a series of alanine peptides with various length from ALA_5_ to ALA_21_ as well as their two derivatives, the (AAQAA)_3_ peptide and a 13-residue KR1 peptide with the formula GN(AAQAA)_2_G, which originated from the (AAQAA)_2_ peptide. Using such a series of peptides helped us to develop a complete procedure, described below, which is robust and considers potential issues with definitions of relevant parameters. To start with, based on multi-microsecond molecular dynamics simulations at room temperature, we find several kinetic and thermodynamic parameters. Out of these, helix populations (calculated via average number of hydrogen bond) as well as relaxation times calculated from the autocorrelation functions on some typical variables, such as the root-mean-square (distance) deviations from the ideal helix (RMSD), become the key observables for our subsequent calculations of friction coefficients. Following the available literature on simple helical peptides, we concentrate on vibrations/exchanges of neighboring hydrogen bonds (HBs) as the main channels for energy dissipations. We present two approaches: the local one based on average single hydrogen bond vibrations and the global one based all the hydrogen bonds within each studied peptide. For calculations of local friction coefficients, we use the fluctuation–dissipation theorem related to reconfigurations of an averaged single HB within the studied peptide. For calculations of global frictional coefficient, we show that robust friction coefficients for complete folding transitions can be obtained from careful considerations of the histograms of end-to-end distances for structures with particular number of hydrogen bonds. The obtained results show the feasibility of using mechanical signatures as a probe of structural changes in the studied peptides. The values of friction coefficients have been found to depend on length of a given peptide as well as details of its folded structure, such as its helical propensity. Local friction coefficients, which varied from 0.06 (ALA_5_) to 2.54 μg/s (ALA_21_), displayed smaller variations among peptides than their global counterparts, which changed between 1.6 (ALA_5_) and 83.8 μg/s (KR1). All these values were obtained at simulated conditions mimicking typical experimental conditions, i.e., water at its standard viscosity and at 0.15 M ionic strength.

## Materials and Methods

### Peptides and Their MD Simulations

Three types of model alanine-based peptides with well-known helical propensities have been considered in this work: (i) alanine-only peptides with 5, 8, 15, and 21 alanine residues, (ii) the KR1 peptide, and (iii) the (AAQAA)_3_ peptide.^4,24^ The KR1 peptide is a derivative of the (AAQAA)_2_ peptide. It was built from 13 residues according to a following formula: H_2_N-GN(AAQAA)_2_G-CONH_2_. Herein, asparagine (N) at the vicinity of the N-cap position and glycine (G) at the C-cap position reflect the highest helical propensities of asparagine and glycine at these positions.^25^ Amide termination of the free carboxyl group prevented appearance of a charged acid group and increased the likelihood of observing hydrogen bond formation.

**Figure 1:**
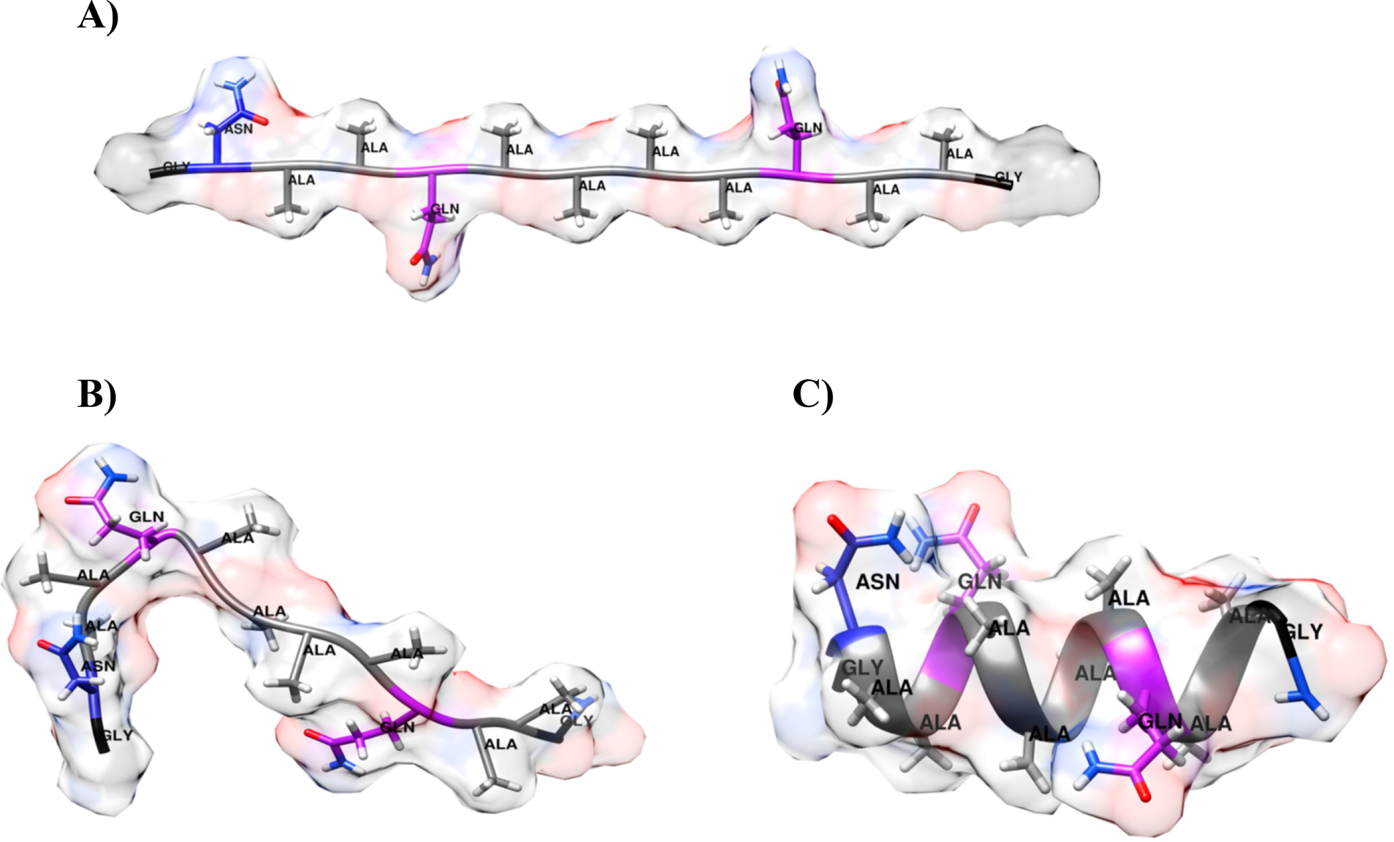
Structures of the KR1 peptide used in MD simulations. (a) Initial highly extended structure before pre-equilibration. (b) Starting structure for the trajectory “e”, i.e., after initial pre-equilibration; (c) starting structure for the trajectory “h”, i.e., an ideal α-helix.

Two 10 μs long molecular dynamics (MD) simulations generated the folding trajectory of the KR1 peptide. These simulations were carried out using GROMACS 5.1.4. software with the CHARMM36m force field, similarly as in ref.^4^ They differed in initial peptide structure. In the first case, an extended KR1 structure has been used to start with; see Figure 1a,b. It was pre-equilibrated first at 100 ns as in ref (4). A cube of edge 4.26 nm with 2538 TIP3P water molecules was used to accommodate a pre-equilibrated extended structure. In the second case, the trajectory was started from an artificially generated ideal α-helix, see Figure 1c. A cube of edge 4.21 nm with 2478 TIP3P water molecules was used to accommodate the α-helical structure. Each system was neutralized by additions of Na^+^ and Cl^–^ ions until an ionic strength of 0.15 M was reached. Before starting the actual MD simulations each system, i.e., a solvated peptide within a simulation box, was briefly equilibrated first with harmonic restraints and then without restraints under *NPT* conditions of 1 bar and 300 K. Trajectory snapshots were saved every 1 ps (extended configuration) or 2 ps (ideal α-helix). Nonbonded cutoffs were 1.2 nm, and the PME method^26^ was used to account for long-range electrostatic interactions. The same approach was taken toward all other studied peptides. Their system compositions along with chosen sizes of the simulation cubic boxes are presented within the Table S1. For each sequence two trajectories were generated: one from the extended (“e”) another one from α-helical (“h”) configuration; see Figures S1–S5. Time lengths of the MD trajectories were as follows: KR1, 2 × 10 μs; ALA_21_, 2 × 20 μs; ALA_15_, 2 × 10 μs; ALA_8_, 2 × 10 μs; ALA_5_, 2 × 5 μs; and AAQAA_3_, 2 × 10 μs.

### Hydrogen Bonds (HBs) and Average Helix Population

Helical HBs refer to hydrogen bonds between an oxygen atom from a carbonyl group C=O of residue “*i*” and a nitrogen atom from an amide group of residue “*i* + 4”. They were considered to be present when an O···N distance between the residues fell below 3.6 Å. All tentative residue pairs were considered. The KR1 peptide has a maximum number of helical hydrogen bonds (MAXHB) of 10, with 9 HBs within the helix and HB 10 at the C terminus. The (ALA)_*n*_ peptides have MAXHB values of 3, 6, 13, and 19 in the cases of ALA_5_, ALA_8_, ALA_15_, and ALA_21_, respectively.^4^ The (AAQAA)_3_ peptide has a MAXHB of 13. A particular number of hydrogen bonds within the *i*th simulated structure (*hb*_*i*_) and the respective values of MAXHB yielded the average helix population, *p*_h_, in a given simulation with the overall number of conformations of *N* through the following formula:
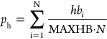
1

For comparative purposes we also used two other methods of obtaining a helical content. One of these methods, denoted by PP, was based on the fraction of residues with helical backbone conformation. Here, a residue was considered in the helical region of the Ramachandran map if its backbone dihedral angles were within 20° of the ideal helix conformation, (ϕ, ψ) = (−62°, −41°). Another method was the DSSP algorithm. The DSSP assigned helical structures using purely electrostatic interactions and atomic coordinates of constituting atoms following the rules initially described in the ref.^27^ implemented in Gromacs.

Finally, a typical distance of 0.15 nm along a helical symmetry axis (*z*-axis) between the subsequent residues within a folded α-helix (a.k.a., helix rise) has been used.^1^

### Correlation Functions

The autocorrelation function, *C*_1_(*t*), revealing how the correlation between the data within generated trajectories of variable *x* have changed as a function of time *t*, was calculated according to a following equation:
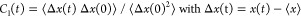
2The angled brackets ⟨...⟩ in eq 2 denote trajectory averages. In this work the variable *x*(*t*) relates to RMSD values, which measure temporal departure of all the backbone C_α_ from a respective ideal α-helix constructed for each peptide.

## Results and Discussion

First, we will present briefly the key MD results obtained for our studied peptides with a particular emphasis on the KR1 peptide, for which the results are presented in the main paper for the first time. Second, we will use the presented MD results to derive several estimates for the friction coefficients of the studied peptides.

### Visualizing Kinetic and Thermodynamics Aspects of Folding of the KR1 Peptide from MD Simulations

To start discussing kinetic aspect of the KR1 folding its two folding trajectories were generated starting from very different initial conditions. Trajectory “e” was initiated from an extended structure, and trajectory “h” was initiated from an ideal α-helix (see Figure 1 and the “Materials and Methods” section). Such an approach was taken to address convergence of the MD simulations, which is considered to be good enough, when at least two independent trajectories explore similar conformational spaces. The same approach with two independently generated MD trajectories was taken for all the other studied peptides. Their respective starting extended (“e”) and α-helical (“h”) configurations are plotted in Figures S1–S5.

**Figure 2:**
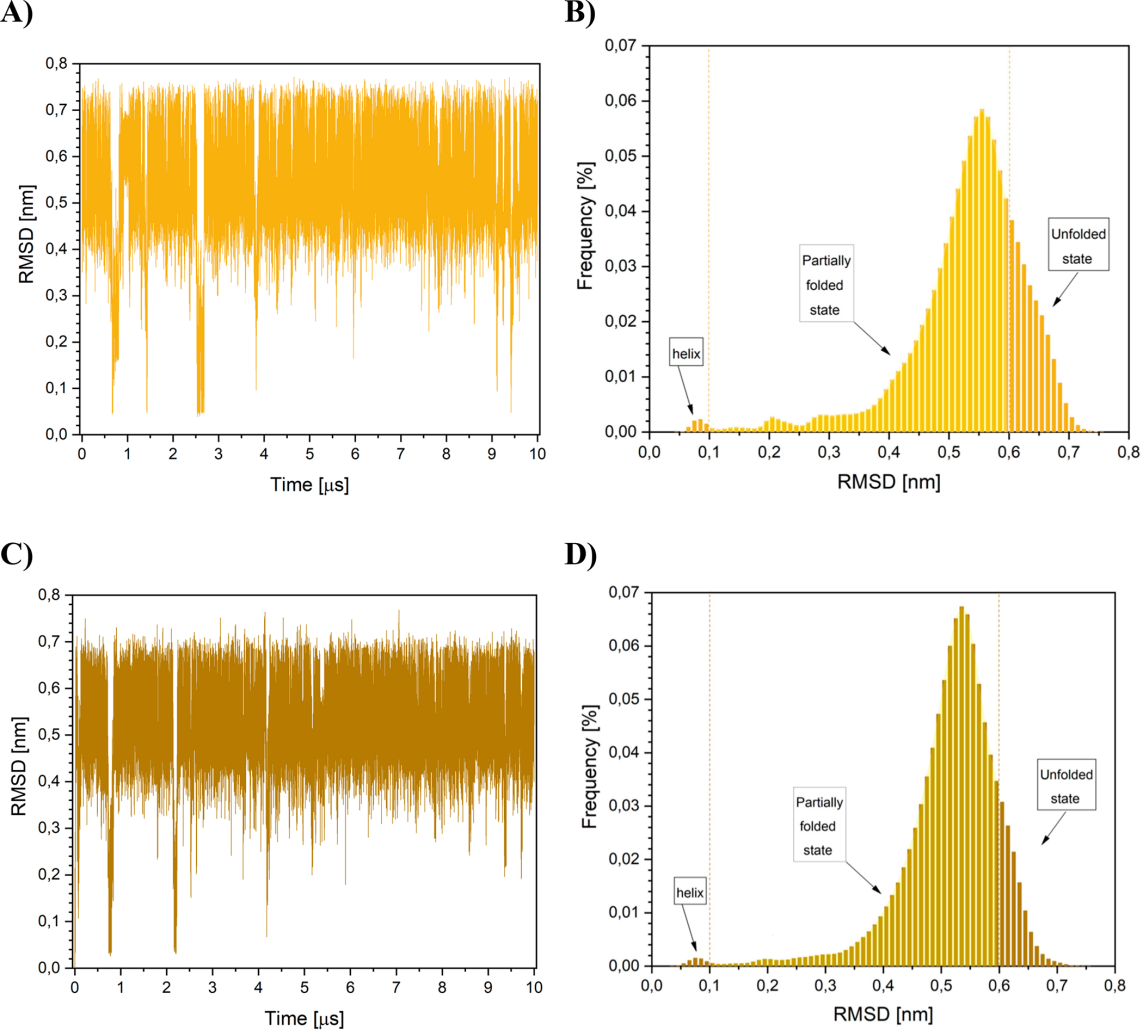
RMSD values for the two probed trajectories of the KR1 peptide. (A) RMSD vs time for trajectory starting from an extended peptide structure (trajectory “e”). (B) Histogram of (A). (C) RMSD vs time for trajectory starting from an α-helical structure (trajectory “h”). (D) Histogram of (C). On the histograms in (B) and (C) a tentative fully folded state has been arbitrary labeled “helix”.

Using the trajectories “h” and “e”, several standard variables have been calculated, including the variations of RMSD of alpha-carbon atoms from an ideal helix with time. Despite different starting configurations, histograms of the RMSD results for the two trajectories of the KR1 peptide are similar to each other; see Figure 2. Therein, folding events characterized by sudden decreases of the RMSD values are rare, and structures with RMSD values below 0.1 nm make up less than 1% of all probed conformations. RMSD trajectories and their histograms indicate clearly that fully folded α-helical forms do not last long and are not highly populated. A great majority of the structures, with RMSD values of more than 0.3 nm, correspond to nonfolded configurations. Substantial overlap among the individual trajectories, observed quite well in the respective histograms in Figures 2b,d suggest that we sampled very similar conformational space and the trajectories were convergent. The RMSD values will be used later to obtain correlation times related to the rate of folding. MD results showing the RMSD data for the ALA_*n*_ peptides have been previously published in the ref (4). The RMSD results for the (AAQAA)_3_ peptide are published in this contribution for the first time in Figure S6.

As another proxy to visualize kinetic folding transitions the trajectories of the end-to-end distance between N- and C- termini of the KR1 peptide were obtained as well as their histograms, see Figure S7. In the case of the KR1 peptide the end-to-end distance is the distance between positions of the first and last, i.e., the 13th α-carbon atoms. Figures 2 and S7 show similar results, i.e., quite rare folding transitions, nonlasting fully folded structures, and reasonable convergence between the trajectories generated for the two different initial conditions. Notably, while the plots of RMSD and end-to-end distances versus time are informative in visualizing conformational transitions, they are not good probes to identify correctly folded and unfolded states. This is because low RMSD values do not encompass all well-folded structures and same values of end-to-end distances correspond to folded and unfolded species.^4^

**Figure 3:**
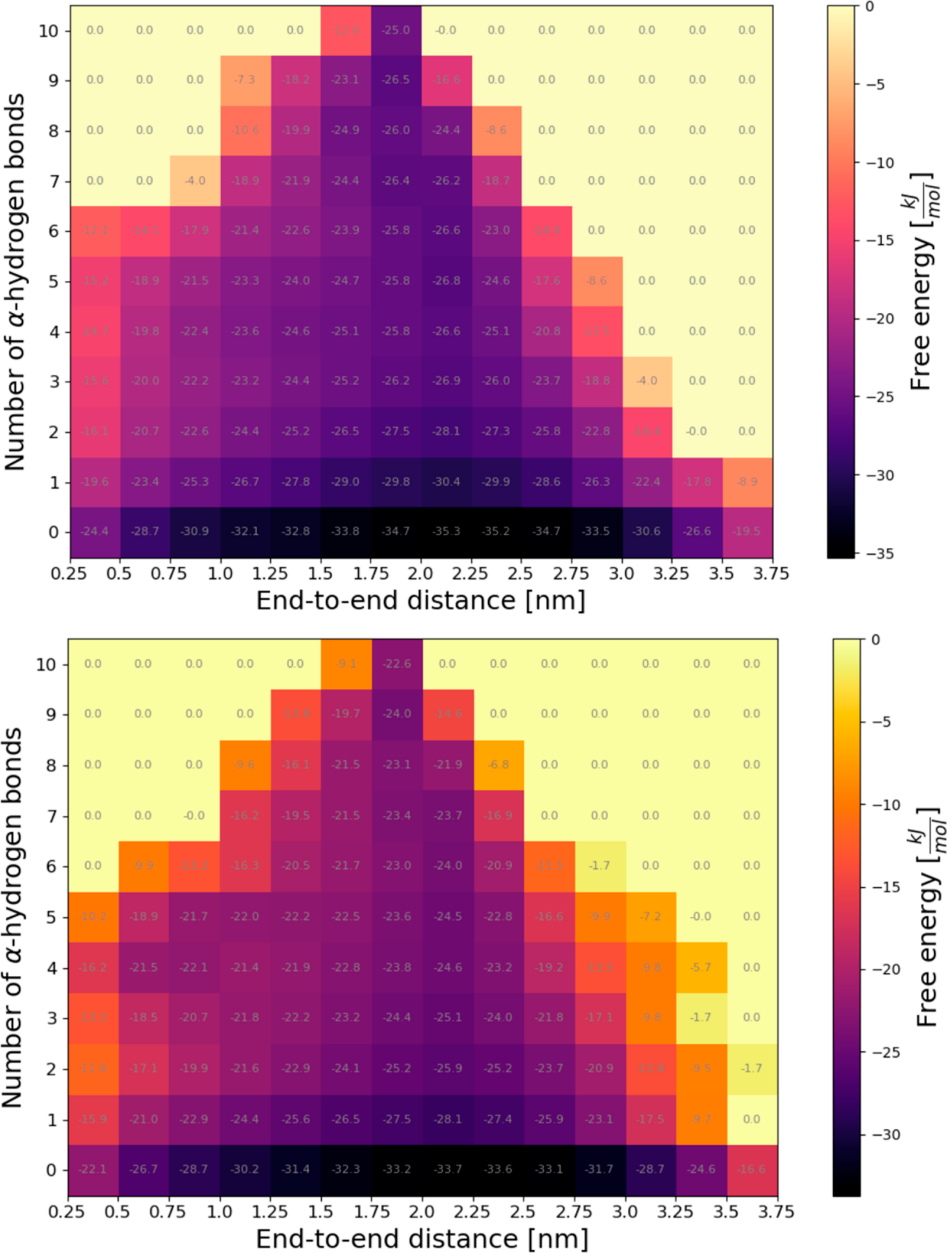
3D plots of number of hydrogen bonds vs end-to-end distance vs apparent free energy Δ*G* = −*RT* ln(no. of conformations) coded by a color. (A) Results for trajectory “h”. (B) Results for trajectory “e”. With 0.15 nm per residue within H-bonded ideal α-helix, the KR1 peptide (13 residues) attains a perfectly folded structure at a distance of 1.95 nm with 10 hydrogen bonds.

In a more comprehensive approach to characterize thermodynamic aspects of the KR1 peptide folding, the folding funnel has been generated via the 3D plots of the number of HBs versus end-to-end distance and apparent free energy of folding Δ*G* as a third coordinate. Figure 3 show the respective plots for the two trajectories, where Δ*G* is color-coded. The number of hydrogen bonds in each structure was obtained according to the standard rules presented in the “Materials and Methods” section. The free energy of folding has been calculated as Δ*G* = −*RT* ln(no. of conformations), where *R* is the ideal gas constant, *T* is absolute temperature, and (no. of conformations) refers to the number of conformations within a given bin, i.e., for a fixed HB number and within a certain range of end-to-end distances.

The data in Figure 3 shows clearly that fully folded KR1 species with a maximum of 10 HBs and end-to-end distances of 1.95 nm (= 13 aa × 0.15 nm) are in the minority. The majority of the results fall within a range of end-to-end distances between 0.75 and 3.0 nm (trajectory “h”) and between 1.25 and 3.0 nm (trajectory “e”). These structures are with zero or one HBs; thus, clearly, they must correspond to the unfolded states.

In addition to MD simulations, the AlphaFold 2 deep mind AI algorithm has been applied as an additional means to obtain the preferential conformations of the folded KR1. This algorithm has been recently made available to the public and its speed in obtaining good quality results has been well acclaimed. The AlphaFold results are presented in the Supporting Information; see Figure S8. Interestingly, a helical structure of the folded KR1 peptide obtained from MD simulations are confirmed by the AlphaFold results. More discussion is presented in the Supporting Information.

### Calculating Selected Kinetic and Thermodynamic Parameters Describing Folding of the KR1 Peptide as Well as Other Studied Peptides

#### Equilibrium Constant

To start, we calculate the equilibrium constant, *K*, for the folding reaction using the folded fraction of the peptide, *p*. By definition the value of *K* is the ratio of respective concentrations of the folded and unfolded species, which is the same as the ratio of their folded and unfolded fractions, i.e., *p* and (1 – *p*), respectively. At the same time, folding is often described via a typical two-state model with *k*_1_ and *k*_–1_ being the folding and unfolding reaction rate constants, respectively. From typical assumptions of folding and unfolding being first-order chemical reactions, the value of *K* equals then to the ratio of *k*_1_ over *k*_–1_. To summarize:
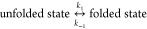
3
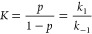
4

Next, we obtain the values of *p* for our peptides from assessments of their helical (i.e., folded) content. There are several standard measures of helical content in MD trajectories that are consistent with experimental data. Counts of helical hydrogen bonds (HB) involving amide C=O groups correspond to the most popular IR experiments that measure secondary structure from shapes of amide absorption bands.^28^ Counts of backbone dihedral values in the helical region of Ramachandran map (PP) correspond to far-UV circular dichroism measurements of secondary structure, which are sensitive to the backbone conformation.^29^ Finally, there exists the DSSP algorithm, which estimates electrostatic interactions between amide groups is often used to analyze secondary structure in X-ray/NMR structures.^27^ The *p* values obtained by each of these methods are presented in the Table 1. See the “Materials and Methods” section for relevant calculation details.

**Table 1:** Fraction of Alpha-Helical Conformations Measured by α-Helical Hydrogen Bonds (HB), Backbone Dihedrals (PP), and DSSP Algorithm^a^

system	HB	PP	DSSP
ALA_5_ h	0.026 ± 0.006	0.090 ± 0.005	
ALA_5_ e	0.032 ± 0.006	0.096 ± 0.004	
ALA_5_ h+e	0.029 ± 0.004	0.093 ± 0.003	
ALA_8_ h	0.060 ± 0.021	0.117 ± 0.019	0.04
ALA_8_ e	0.068 ± 0.020	0.122 ± 0.016	0.05
ALA_8_ h+e	0.063 ± 0.014	0.119 ± 0.012	0.05
ALA_15_ h	0.219 ± 0.132	0.254 ± 0.112	0.21
ALA_15_ e	0.278 ± 0.116	0.306 ± 0.099	0.27
ALA_15_ h+e	0.249 ± 0.087	0.280 ± 0.075	0.24
ALA_21_ h	0.590 ± 0.099	0.584 ± 0.085	0.58
ALA_21_ e	0.585 ± 0.151	0.578 ± 0.131	0.60
ALA_21_ h+e	0.588 ± 0.090	0.581 ± 0.078	0.59
(AAQAA)_3_ h	0.236 ± 0.099	0.270 ± 0.086	0.23
(AAQAA)_3_ e	0.198 ± 0.083	0.237 ± 0.073	0.19
(AAQAA)_3_ h+e	0.217 ± 0.065	0.254 ± 0.057	0.21
KR1 h	0.046 ± 0.023	0.056 ± 0.027	0.06
KR1 e	0.057 ± 0.030	0.070 ± 0.039	0.04
KR1 h+e	0.052 ± 0.018	0.063 ± 0.020	0.05

a See the “Materials and Methods” section for details. Averaged results for the two probed trajectories in the case of each peptide are highlighted in light gray. ALA_*n*_ results are from ref (4).

The results of helical content from Table 1 are very similar for most of the peptides across all used methods except the smallest peptides, ALA_5_ and ALA_8_. Therein, the HB methods yields smaller values of helicities than the PP method. Quite likely the reason is that the PP method is more inclusive than the HB count, which shows particularly well in the case of smaller and labile peptides. Therefore, the HB count method will be used to obtain the *p* values for the studied peptides. To do so, we will use eq 1 with *p*_h_ = *p*. To account on heterogeneity of the MD results an averaged result for the two studied trajectories will be reported, as in Table 1. Consequently, for the KR1 peptide the value of *p* = *p*_h_ = 0.052 ± 0.018 will be considered for further calculations of relevant parameters.

Keeping in mind that KR1 peptide is a derivative of (AAQAA)_2_ peptide, its ca. 5% helical content is much lower than that in the cases of peptides with comparable length such as ALA_8_, ALA_15_, and (AAQAA)_3_ peptides, where *p* values between 6 and 25% were obtained. However, the presence of a Gly residue on both ends as well as lack of a blocking group at its N-terminus seem to be at least partially responsible. Interestingly, similar factors contributed to lower number of helical conformations assumed by Gly residues in the AlphaFold results; see the Supporting Information.

Finally, helical content can also be obtained from the RMSD plots.^4^ For example, using the RMSD histograms from Figure 2 for the KR1 peptide, the value of *p* was arbitrary estimated by counting the states with RMSD values between zero and 0.1. Such states are labeled “helix” in Figure 2. The RMSD results for the two trajectories yield an average *p* of only 0.006 ± 0.008, which is almost an order of magnitude lower than the values in Table 1. Therefore, the RMSD method is deemed here as the one seriously underestimating a helical content and will not be used.

Using the values of *p* obtained from the HB method, the equilibrium constant, *K*, are obtained from eq 4. In the case of the KR1 peptide, one gets: *K* = 0.055 ± 0.020. In the case of other peptides, please refer to the Table S2, which lists as well free energy change associated with folding and calculated as Δ*G* = −*RT* ln(*K*). The obtained values of *K* and Δ*G* for the KR1 peptide confirm that it prefers strongly to stay in unfolded configurations (*K* ≪ 1, Δ*G* = 1.73 ± 0.22 kcal/mol). Similar conclusions can be drawn for the small and labile ALA_5_ and ALA_8_ peptides. However, all other peptides prefer to be more folded, with their respective values of *K* getting closer to unity and even exceeding unity in the case of the ALA_21_ peptide.

#### Reaction Rate Constants

In the next step, reaction rate constants are obtained from equilibrium constants and correlation times of the MD trajectories. When considering folding as a first order chemical reaction, the values of folding (f) and unfolding (u) reaction rate constants are directly related to their respective time constants τ_1_ and τ_–1_ via: *k*_1_ = 1/τ_1_ and *k*–1 = 1/τ_–1_. The time constants τ_1_ and τ_–1_ are, in turn, related to the correlation times, τ_c_, obtained from the correlation function of the folding trajectories via: 1/τ_1_ + 1/τ_–1_ = 1/τ_c_.^4^ Using such considerations and eq 4 one obtains:
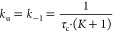
5a
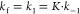
5b

**Figure 4:**
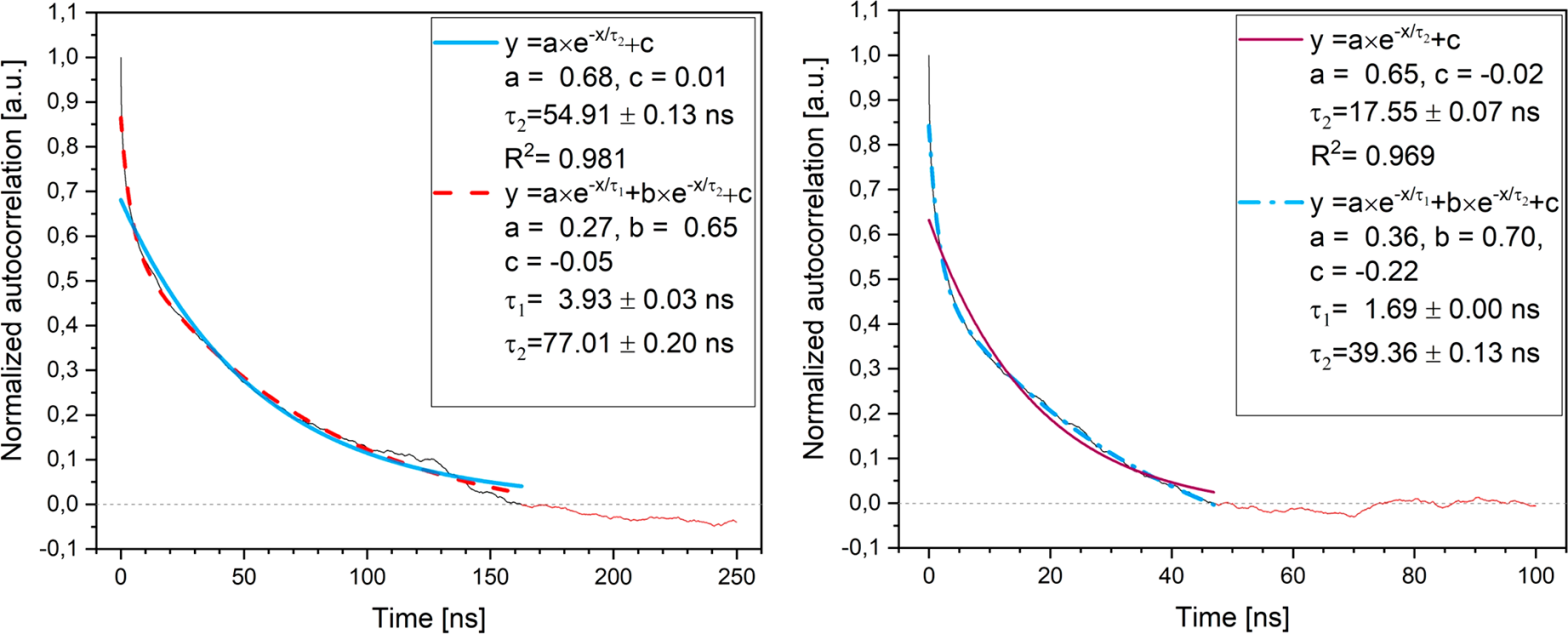
Correlation times for the KR1 peptide obtained from RMSD data for (left) trajectory “e”, and (right) trajectory “h” after performing a double exponential fitting.

In the case of the KR1 peptides, the times τ_c_ are obtained from the changes of the RMSD for the trajectories “e” and “h” in Figure 2, since this is one of common measures of the folding events.^4^ The normalized autocorrelation functions of these RMSD distances have been calculated using eq 2 and are plotted in Figure 4.

The data in Figure 4 were fitted with mono- and two-exponential decays, since autocorrelation functions are often regarded as a series of exponential terms with their appropriate weights.^4^ While the use of a double exponential is empirical, both visually and via χ^2^ tests, the two-exponential decay fits the data much better and such results will be considered. More details are presented in Tables S3 and S4 and Figures S9 and S10. Visibly different relaxation times among “e” and “h” simulations for the KR1 peptide are indicators that the simulations did not converge perfectly. Nevertheless, as before, to account on the heterogeneity of the obtained results, averaged values of the shorter and longer correlation times are reported. Such averaged values for the KR1 peptide are τ_c1_ = τ_c1,ave_= 2.81 ± 1.12 ns and τ_c2_ = τ_c2,ave_ = 58.18 ± 18.83 ns. Better agreement between “e” and “h” simulations was found in the case of the (AAQAA)_3_ peptide, where we obtained τ_c1_ = τ_c1,ave_= 0.77 ± 0.01 ns and τ_c2_ = τ_c2,ave_ = 97.0 ± 9.8 ns. The results of the correlations times for the (ALA)_*n*_ peptides were previously published. Due to imperfect convergence of the simulations in the case of ALA_15_ and ALA_21_, the values for local MD ACF (autocorrelation function) RMSD fits will be used for the (ALA)_*n*_ peptides, see Table S5 for a complete set of values. Overall, Table 2 lists the correlation times and their errors together with respective folding and unfolding rate constants (obtained and discussed later) for all peptides.

**Table 2:** Correlation Times (from RMSD ACF Fits) and Corresponding Values of the Folding and Unfolding Rate Constants (from eq 6) for All the Peptides Investigated Here

system	τ_c1_ (ns)	τ_c2_ (ns)	*k*_–1_ (μs^–1^)	*k*_1_ (μs^–1^)
ALA_5_ h+e	0.15 ± 0.05	2.00 ± 0.10	486 ± 26.3	14.5 ± 2.8
ALA_8_ h+e	0.83 ± 0.13	12.5 ± 0.5	75.0 ± 4.12	5.04 ± 1.47
ALA_15_ h+e	1.80 ± 0.10	88.5 ± 11.5	8.49 ± 2.09	2.81 ± 2.00
ALA_21_ h+e	6.90 ± 0.10	185 ± 15	2.23 ± 0.67	3.18 ± 2.13
(AAQAA)_3_ h+e	0.77 ± 0.05	97.0 ± 11.0	8.07 ± 1.59	2.24 ± 1.30
KR1 h+e	2.81 ± 1.12	58.2 ± 18.8	16.3 ± 5.6	0.89 ± 0.63

Based on analysis of the MD trajectories, the shorter of the correlation times, τ_c1_, may be associated with length fluctuation for individual hydrogen bonds.^4^ Relaxations of this type, on the 20 ns time scale have been detected experimentally for a model 21-residue peptide WH21.^23^ The presence of such fast relaxations has also been predicted from a theoretical standpoint, with τ_c1_ expected to correspond to the fastest detectable dissipative vibrations in the system, involving single hydrogen bonds and/or associated waters of hydration.^30^ Thus, τ_c1_ are expected to relate to an average time necessary for a single hydrogen bond breaking/formation, and we use this time to describe hydrogen bond migration within an α-helix of a given length. From this perspective such times may be expected to be similar for all the alanine based α-helical peptides. Indeed, the values of τ_c1_ in Table 2 appear to be on the order of 0.1 ns to several nanoseconds and increase relatively slowly with length of the helix, unlike the folding times, which vary more strongly. Interestingly, for the KR1 peptide, which is 13 residues in length, the τ_c1_ time is higher than for the ALA_15_ peptide, but in the case of an equivalent (AAQAA)_3_ it is smaller than for the ALA_15_ peptide. Taking into account that ALA_15_ and (AAQAA)_3_ have identical length and similar helical contents of 25 and 24% (see Table 1), one would expect similar τ_c1_ values. However, this is not the case, since τ_c1_ values of ca. 0.8 and 1.8 ns are obtained for (AAQAA)_3_ and ALA_15_, respectively. Thus, it appears that not only peptide length but also specific sequence details are important for determination of the τ_c1_ values. It must be noted that values of τ_c1_ also depend on the choice of considered variable (RMSD or other), trajectory length, and fitting details.

The longer correlation time, τ_c2_, differs substantially across various peptides. It has been associated with the time necessary for a complete helix formation,^4^ and indeed helix formation times do not scale linearly with the helix length. Therefore, the values of τ_c2_ will be utilized to obtain folding and unfolding rate constants, which from eq 5 are calculated as
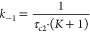
6a
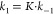
6b

Applying eq 6, in the case of the KR1 peptide one obtains: *k*_–1_ = 16.3 ± 5.6 MHz and *k*_1_ = 0.89 ± 0.63 MHz. The complete results for all studied peptides were presented in the Table 2 above. Remarkably, only the ALA_21_ peptide has more than 50% of helical content under simulated conditions (see Table 1), and only in this case was the folding rate constant (*k*_1_) higher than the unfolding rate constant (*k*_–1_).

### Derivation of Friction Coefficient for KR1 and Other Studied Peptides in PBS

Herein we will present two approaches, which we call “local” and “global” estimates of friction coefficient encompassing internal and solvent-induced friction. To start, we obtain relationships describing such friction coefficient within the studied peptides. For such a purpose we will use the Einstein relation, which is a form of fluctuation–dissipation relation and relates an appropriate self-diffusion coefficient, *D*, within a molecule with an associated friction coefficient, γ. Following the Stokes model, the value of γ can be regarded as a proportionality factor in a frictional force −γ × υ originating from the Brownian motion of molecular fragments with the velocity υ. Velocity-dependent friction and fluctuation–dissipation formulas have found their use across many branches of physical chemistry, in particular in describing the motion of a Brownian particle via a Langevin equation, i.e., an equation describing motion of the particle subjected by a frictional force and an additional intrinsic “fluctuating” force.^19^ By analogy, in our local approach to estimate the friction we will consider diffusion and Brownian motion of a single hydrogen bond within an α-helix as the main source of its energy dissipations. Via such consideration, one arrives at a straightforward interpretation of the Einstein–Stokes relation obtained in the equilibrium limit:
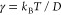
7where *k*_B_ is the Boltzmann’s constant, *T* is the absolute temperature, and *D* is the diffusion coefficient of a single hydrogen bond within the helix. Based on the mathematical properties of the Brownian motion along a generalized one-dimensional variable describing HB extension the value of *D* can be calculated as^19^
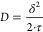
8where δ = 0.15 nm is the “single diffusion step” considered here as the helix rise (see the “Materials and Methods” section), and τ is the necessary reconfiguration time for a single hydrogen bond to switch between pairs of neighboring residues along an α-helix. Based on the discussion of the correlations times in the previous section, the value of τ can straightforwardly relate to the MD simulations. It be calculated as an autocorrelation time associated with switching a HB between neighboring residues within an α-helix, i.e., τ = τ_c1_. Thus, from eqs 7 and 8 and using τ_c1_, one gets a local estimate of the dissipation factor, γ_L_, within a folded peptide, γ = γ_L_. In the case of the KR1 peptide one gets the values of *D* = (4.0 ± 1.6) × 10^–12^ m^2^/s and γ_L_ = (1.03 ± 0.41) × 10^–9^ kg/s. The values of *D* and γ_L_ for all studied peptides are listed in the Table 3 below.

**Table 3:** Summary of the Obtained Values Related Friction Coefficients for the Helical Peptides Studied Here^a^

system	*D*(10^–12^ m^2^/s)	γ_L_(10^–9^ kg/s)	Δ*x* (nm)	γ_*K*1_(10^–9^ kg/s)	γ_*K*2_(10^–9^ kg/s)
ALA_5_	75.0 ± 25.0	0.06 ± 0.02	0.5223 ± 0.0039	1.08 ± 0.02	1.6
ALA_8_	13.6 ± 2.1	0.30 ± 0.05	0.6423 ± 0.0069	2.13 ± 0.05	4.7
ALA_15_	6.3 ± 0.3	0.66 ± 0.04	0.0158 ± 0.0164	7,852 ± 16,301	27.9
ALA_21_	1.6 ± 0.10	2.54 ± 0.04	–0.5284 ± 0.1374	11.3 ± 5.9	29.9
(AAQAA)_3_	14.6 ± 0.9	0.28 ± 0.03	–0.0153 ± 0.0178	10,101 ± 23,503	39.9
KR1	4.0 ± 1.6	1.03 ± 0.41	0.329 ± 0.021	45.2 ± 5.7	83.8

a Data averaged for “h” and “e” simulations. Diffusion constants, *D*, were calculated for local HB diffusion of HB via eq 8. Using such values of diffusion constants, local values of friction, γ_L_, have been calculated using eq 7. Finally, global values of friction were calculated via eq 9 and using associated values of Δ*x* obtained from the data in Figure 6; see text.

Nearly all values of γ_L_ in Table 3 range between 0.3 and 2.5 μg/s except of the smallest ALA_5_ peptide. It becomes then clear that local friction is related to peptide length and sequence details. The rough trend is set for ALA peptides, where longer peptides exhibit higher helix content *p* and have higher local friction. Furthermore, the (AAQAA)_3_ peptide, with apparently improved helical propensity with respect to its ALA_15_ homologue, has notably lower local friction than the ALA_15_. However, the KR1 peptide is shorter than ALA_15_ and (AAQAA)_3_ has substantially higher friction. Thus, we hypothesize that local friction is correlated with helix stability and helix propensity. Such a hypothesis appears to be a reasonable expectation from the point of view of mechanical systems. However, further studies in homologous series of mutated ALA peptides are needed to confirm this observation and to explore specific microscopic effects of various side chain substitutions.

Besides a very local approach, related to local exchanges/fluctuations of individual hydrogen bonds, we also derive a global approach to friction associated with folding/unfolding transitions. This approach will be based on the model of Khatri et al.^20^ who considered defined hops, Δ*x*, in the length of the molecule between microstates separated by a simple energy barrier and with the reaction rates *k*_1,m_ and *k*_–1,m_ for the forward and reverse reactions, respectively. In such a case, Khatri et al. obtained that friction coefficient, called here later γ_*K*1_, could be described by the following equation:
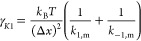
9

Equation 9 has been obtained from the Langevin equation, by a formalism similar to that of eq 7.^20^ Upon a quick consideration it reduces to eq 7 when Δ*x* is substituted by δ and inverses of the respective rate constants are substituted by respective forward and backward diffusion times for single HB.

Beyond local HB diffusion treated in the previous section, eq 9 can be interpreted as the energy dissipated within conformational change between any two microstates of the system provided that the corresponding Δ*x* and given reaction rate constants are known. With this respect one can calculate a “global” friction coefficient related to the transition between unfolded and folded states by associating the values of *k*_1,m_ and *k*_–1,m_ from eq 9 with *k*_1_ and *k*_–1_ from eq 6. The key detail for such calculations of “global” friction coefficient is to find out a respective value of Δ*x*. A first take would suggest to use an end-to-end distance change within the peptide associated with a transition from unfolded to folded ensemble of states. However, considered here peptides are quite short and similar distances usually correspond to both folded and unfolded species. In contrast, end-to-end distances for populations with a particular number of hydrogen bonds are expected to provide much better estimates of a relevant Δ*x*. Such an approach follows up our earlier calculation of “local” friction, which was also based on hydrogen bonds.

**Figure 5:**
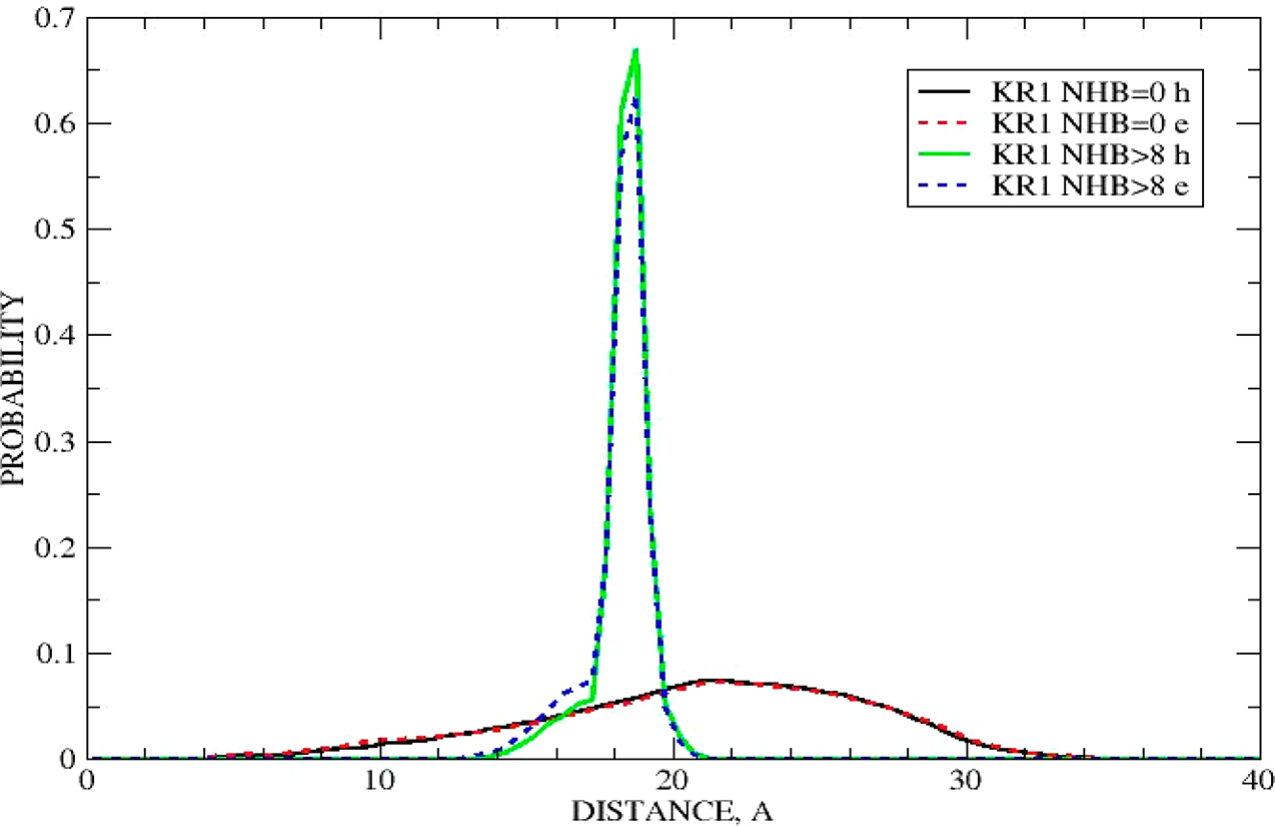
Histograms of end-to-end distances corresponding to a selected number of hydrogen bonds in the KR1 peptide for “e” and “h” trajectories.

The ideal helix form of the KR1 peptide exhibits 10 α-helical hydrogen bonds. For the purposes of calculating the length change upon unfolding, we define the KR1 folded state as the state with 9 or 10 α-helical HBs, and the unfolded state as the state with 0 HBs. From Figure 5 one obtains that an end-to-end distance corresponding to a population of such folded states with 9 or 10 HBs averaged over the two used trajectories is at *x*_1_ = 1.864 ± 0.002 nm at 95% confidence intervals. An unfolded state with no HBs, i.e., a totally disrupted peptide, is very broad with a large value of the full width at half height (fwhh). Nevertheless, due to a huge amount of data, its maximum is set at *x*_2_ = 2.193 ± 0.019 nm at 95% confidence intervals. Correspondingly, a value of Δ*x* associated with a complete unfolding transition corresponds to Δ*x* = *x*_2_ – *x*_1_ = 0.329 ± 0.021. Applying eq 9 to this data yields γ_*K*1_= 45.2 ± 5.7 μg/s.

The column γ_*K*1_ in the Table 3 list values of friction coefficients obtained via an aforementioned approach for all studied peptides. The γ_*K*1_ values increase with peptide’s length and are substantially larger than the ones obtained via the local approach. However, such values appear substantially overestimated in the case of ALA_15_ and (AAQAA)_3_ peptides. These problems are related to unusually small values of Δ*x* obtained for these systems between their folded and unfolded states. The distributions of Δ*x* for 0 HB are quite flat, and while their maxima are well-defined, their fwhh are substantial. Errors at 95% confidence intervals are still well-defined due to a huge amount of data, i.e., several million data points. To address whether there are any other reasons for an odd behavior of the 15 residue peptides, we decided to investigate the structures with a given number of HBs in a greater detail.

**Figure 6:**
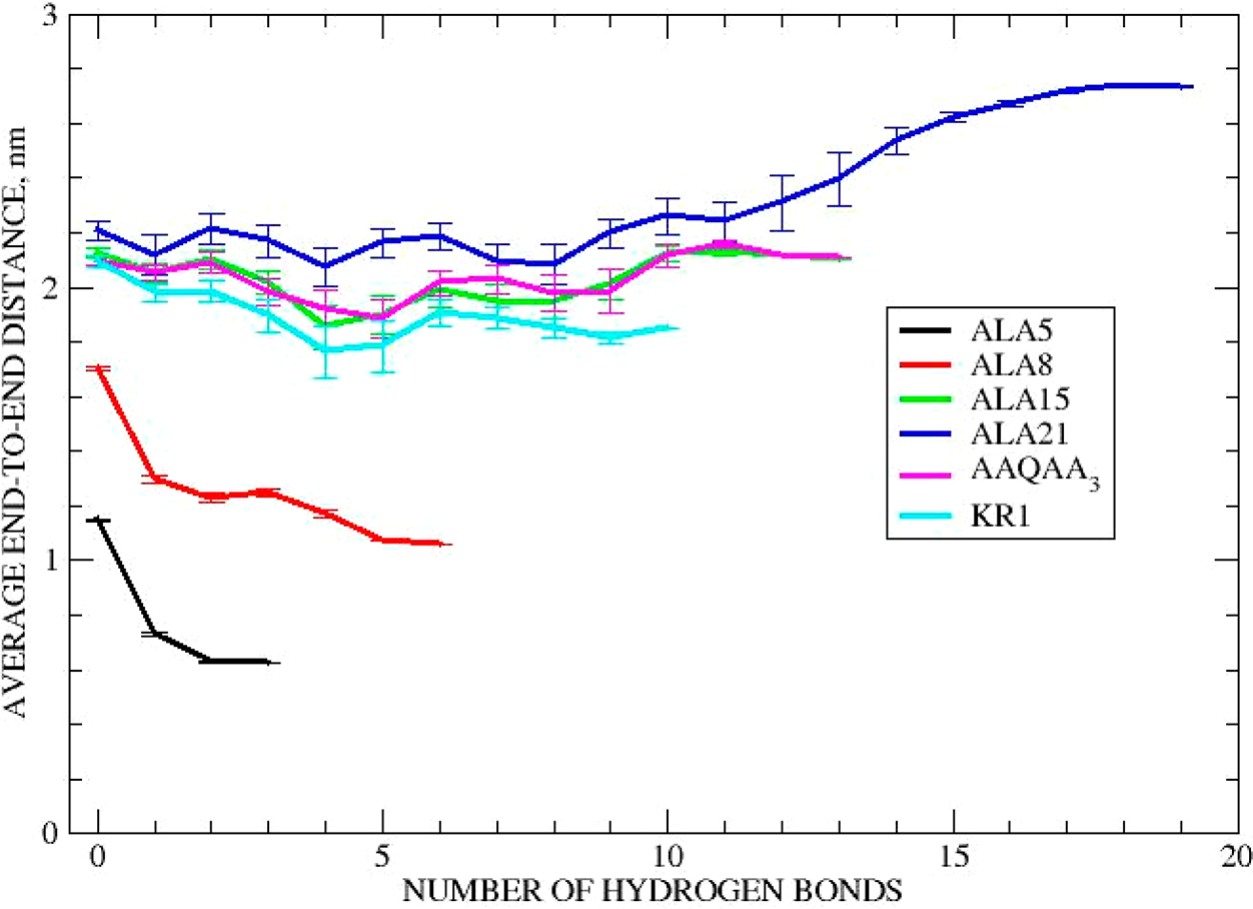
End-to-end distances of peptides averaged over subpopulations with fixed number of hydrogen bonds. Averages over h and e trajectories. Error bars reflect 95% confidence intervals.

Figure 6 plots the averages and standard errors obtained from distributions of end-to-end distances for all studied here peptide structures with a given number of HBs. One can clearly see that the average values exhibit clear trends for ALA_5_, ALA_8_ and ALA_21_, but there is little systematic variation for the remaining peptides of intermediate length. Coincidently, in the cases between 10 to 15 HBs the differences between initial and final maxima become very small. This is exactly the case of ALA_15_ and (AAQAA)_3_ peptides. Therefore, since (Δ*x*)^2^ values appear in eq 9, we decided to sum up all the (Δ*x*_*n*_)^2^ calculated for respective transitions from *n* to *n* + 1 HBs which leads to γ_*K*2_ calculated via eq 10:
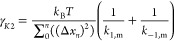
10

Applying eq 10 to our data might overestimate a final result via not properly accounting on the cooperativity in folding, but at least all contributions to friction along a *gedanken* sequential transition from unstructured peptides with zero HB to properly folded peptides with a maximum number of HBs will be taken care of. The γ_*K*2_ results are reported in the Table 3, and the data plotted in Figure 6 are listed in the Supporting Information.

The main success of the presented approach is that the results of “global” friction coefficients γ_*K*2_ are far more robust than γ_*K*1_ since no outliers are found. However, relative errors associated with γ_*K*2_ are huge and extend to several hundred percent. This is because we calculate differences between average end-to-end distances for consecutive populations of HBs, and such differences come out very small and often similar to their errors. The values of γ_*K*2_ are also roughly 2 orders of magnitude larger than the values of “local” friction. We consider here a substantial conformational transition within the molecule, i.e., effectively bringing it from zero to a maximum number of HBs. Thus, substantially larger values of friction coefficients in comparison to γ_L_ are expected, but quantitative comparisons with experiments are needed. The values of γ_*K*2_ increase monotonically with the helix length apart of the KR1 peptide. Considering its size, the KR1 peptide is also very slow to fold, displays very small helical content *p*, and likely has a very small helix propensity.

In summary, the obtained friction coefficients are sensitive measures of conformational transitions within the molecule, and the two presented approaches can be regarded as the two limiting cases. From a continuous mechanics perspective any local breaking/rearrangement of a single hydrogen bond might be seen as a limiting case of purely elastic stretching/compression of a helix, upon which no internal friction would be encountered and no energy dissipated. Then, rupture and reformation of a single HB between the same and/or neighboring (in space) residues appears as the most natural mechanism for energy dissipation and internal friction within a folded helix. Therefore, our estimates of local friction included kinetic parameters related to fluctuation of single HBs via τ_c1_. The obtained values of such friction coefficients, here called “local” friction, have been found to generally increase with peptide length from 0.06 to 2.54 μg/s (= 10^–9^ kg/s) for our mix of 5–21 residue α-helical peptides. We suggest the following interpretation for the “local” friction coefficients. Such friction can be associated with the damping constant in the damped harmonic oscillator. There are virtually no hydrophobic interactions within our studied peptides. Thus, microscopic interpretations for damped vibrations leading the largest energy dissipation include mostly energy lost in vibrations and local exchanges of hydrogen bonds and/or vibrations and local displacements of the coupled water molecules. If true, then this confirms our approach to use HB dynamics as good proxies of local friction within folded α-helical peptides.

Despite currently lacking experimental validation of friction coefficients for studied here peptide, there are several experimental studies with some relevance. To start with, in the case of unfolded proteins, Taniguchi et al. via applying models of damped harmonic oscillators estimated that at low stretching their viscoelastic friction coefficient γ_*n*_ = 2 × 10^–8^ kg/s, while the limit of extended stretching γ_*n*_ increased about 2 times.^10^ These studies attributed the measured results to friction associated with jumps between coiled and uncoiled myosin rods, which involve rupture of many α-helical HBs. From slow stretching of simultaneously vibrating polystyrene polymer chains with AFM Nakajima and Nishi obtained γ_*n*_ = 3 × 10^–9^ kg/s at ∼100–200 pN and 3 × 10^–8^ kg/s at 600 pN tensile forces.^31^ Therein, the measured viscoelastic coefficient was mostly attributed to the monomer–solvent friction. However, no measurements at varying solvent friction were performed in that work. In other experimental approaches, Bippes et al. estimated γ_*n*_ between 10^–7^ kg/s to 10^–5^ kg/s for dextrans stretched with AFM.^16^ Therein, single chair–boat isomerization events within sugar rings were qualitatively associated with observed values of friction coefficients. In the case of vibrations of folded proteins, viscoelastic studies reported γ_*n*_ = 4.4 × 10^–5^ kg/s for guanylate kinase^17^ and γ_*n*_ = 2 × 10^–8^ kg/s for myosin rods.^10^ Finally, an upper bound on the friction coefficient within folded I27 domains (mostly β-type) with 89 residues from titin has been estimated as not exceeding 3 × 10^–7^ kg/s by one study^8^ or 5 × 10^–7^ kg/s by another study.^11^ These would relate to energy dissipations related to vibrations of a much larger structure than considered here peptides and likely its interactions with solvent molecules, which once again are difficult to relate to our present calculations.

Overall, despite being obtained for systems other than peptides and depending on the scale of the probed conformational transitions, the experimental estimates of friction coefficients for molecules under biologically relevant forces fall between 10^–9^ and 10^–6^ kg/s. Despite the values containing the contributions from internal friction and solvent, this is still a good agreement with friction coefficient estimated herein via our local and global approaches.

## Conclusions

In this paper we presented some key results of MD simulations for several helix-forming peptides. New results were described for the (AAQAA)_3_ and KR1 peptides. We also reported complementary data for the ALA_*n*_ peptides with 5, 8, 15, and 21 residues (some of which were previously published). The main novelty of this paper was to develop models and approaches for obtaining estimates of friction coefficients using the MD results. Two kinds of friction coefficient values were obtained based on our hypothesis that, at least in the case of α-helical peptides, processes associated with HB fluctuations and rearrangements provide good measures for major channels of energy dissipations.

First, the “local” friction coefficients has been estimated from the local dissipative vibrations/switching of HBs between neighboring residues along an α-helix. The obtained values of such friction have been found to differentiate very well any differences in the structure and folds of the peptides. For the (ALA)_*n*_ peptides, they increased from 0.06 to 2.54 μg/s (= 10^–9^ kg/s) with peptide length changing from 5 to 21 residues. Departures from this trend were obtained in the case of structurally different peptides, e.g., the KR1 and (AAQAA)_3_ peptides for which values of 1.03 and 0.28 μg/s were obtained. Further analysis prompted us to set a hypothesis that “local” friction for α-helical for systems with the same number of residues is the lowest for a peptide with highest helical propensity. Such a hypothesis, however, needs further verification for a homologous series of same length peptides differing in composition.

We also estimated friction coefficients changes related to larger conformational transitions, such as complete folding/unfolding of a peptide. Such approach led us to estimate the values of “global” friction coefficients based on the model of Khatri et al.^20^ The most promising estimates came out to consider additions to friction coefficients from all individual HBs formation events. We obtained that “global” friction varied between 1.6 and 83.8 μg/s (= 10^–9^ kg/s) for our mix of 5- to 21-residue α-helical peptides. Such friction coefficients differentiated very well any subtle structural changes between the studied peptides. In the homogeneous case of the ALA peptides “global” friction coefficients increased with the helix length, while substantial departures were obtained in the substituted ALA peptides, namely, for the (AAQAA)_3_ and KR1 peptides. Our approach for calculations of the “global” friction coefficients did not take into account cooperativity in folding and therefore can be seen as an upper limit of the expected energy dissipation during the folding process. In addition, the contributions from the solvent were not estimated either.

Since the current experimental results of friction coefficients for helical peptides are lacking, we compared our results with an eclectic mix of friction coefficients results obtained by other authors in the case of various proteins and biomolecules. The comparisons came out to be striking, since results similar to our estimated values of the friction coefficients were reported, i.e., varying between 10^–9^ and 10^–6^ kg/s.

Taking into account that “global” friction is expected to depend on length of the protein as well as details of its folded structure (here only α-helical peptides were studied), one can expect that for larger peptides and proteins values of friction coefficients of more than 10^–6^ kg/s will be obtained. However, since γ_*n*_ depends not only on internal relaxations within the molecule but also on the surrounding medium, it might well be that dependence of friction coefficients on surrounding medium becomes more prominent on the larger structures and will dwarf any dependence on the internal order of HBs. In other words, a crossover to entirely different mechanisms of friction for larger peptides and proteins is not excluded.

## Supporting Information Available

Detailed compositions of the studied systems; initial structures of the peptides for MD simulations; selected structural changes (RMSD, HB count, end-to-end distance) for some peptides along MD simulations; AlphaFold results and their discussion for the KR1 peptides; equilibrium constants and free energy changes for folding of the studied peptides; procedures for obtaining fits to autocorrelation functions obtained from the RMSD data and for obtaining correlation times; the data for obtaining Figure 6 in the main paper (PDF)
