# Supplementary material for: Analytical Approaches for Deriving Friction Coefficients for Selected α-Helical Peptides Based Entirely on Molecular Dynamics Simulations
Source: J Phys Chem B. 2022 Oct 27;126(44):8901–12. doi: 10.1021/acs.jpcb.2c03076 (PMC9661531; doi:10.1021/acs.jpcb.2c03076)
Supplement: Supplementary file 1 — jp2c03076_si_001.pdf [file jp2c03076_si_001.pdf]

Supplementary Materials

Analytical Approaches for Deriving Friction Coefficients for Selected α-helical Peptides and Based Entirely on Molecular Dynamics Simulations.

Aleksandra Wosztyl[1], Krzysztof Kuczera*[,2,3] and Robert Szoszkiewicz*[,1]

1 Faculty of Chemistry, Biological and Chemical Research Centre, University of Warsaw, Żwirki i Wigury 101, 02-089 Warsaw, Poland.
2 Department of Chemistry, The University of Kansas, Lawrence, Kansas 66045, USA.
3 Department of Molecular Biosciences, The University of Kansas, Lawrence, Kansas 66045, USA.

(*) Corresponding Authors: rszoszkiewicz@chem.uw.edu.pl, kkuczera@ku.edu.

1) The system compositions for all the studied peptides.

System	Pep	Tot	Wat	Na+	Cl-	a
ALA5 h	59	2966	967	3	3	3.08
ALA5 e	59	3734	1223	3	3	3.33
ALA8 h	89	4162	1355	4	4	3.44
ALA8 e	89	4585	1496	4	4	3.57
ALA15 h	159	8583	2726	8	8	4.36
ALA15 e	159	8866	2897	8	8	4.45
ALA21 h	219	12077	3942	16	16	4.93
ALA21 e	219	12032	3927	16	16	4.92
$(AAQAA)_3$ h	180	8557	2387	8	8	4.39
$(AAQAA)_3$ e	180	8557	2387	8	8	4.42
KR1 h	147	7596	2478	7	8	4.21
KR1 e	147	7776	2538	7	8	4.26

Table S1. *Pep – peptide atoms, Tot – total atoms, Wat – water molecules, Na+ - sodium ions, Cl- - chloride ions, a – cubic box size, nm. h – trajectory started from helix, e – trajectory started from extended conformation. ALA_n data are from Ref.*[1]

2) Initial structures for the MD trajectories.

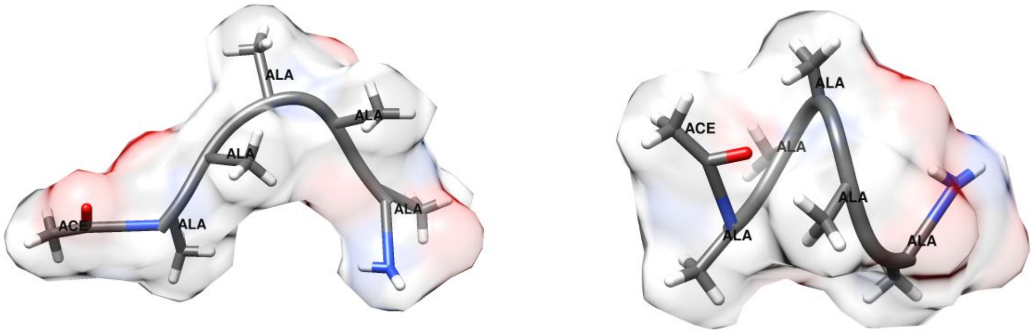

Figure S1. *The initial structures of the ALA_5 peptide used in simulations. Left: an extended structure ("e"), right an alpha-helical structure ("h").*

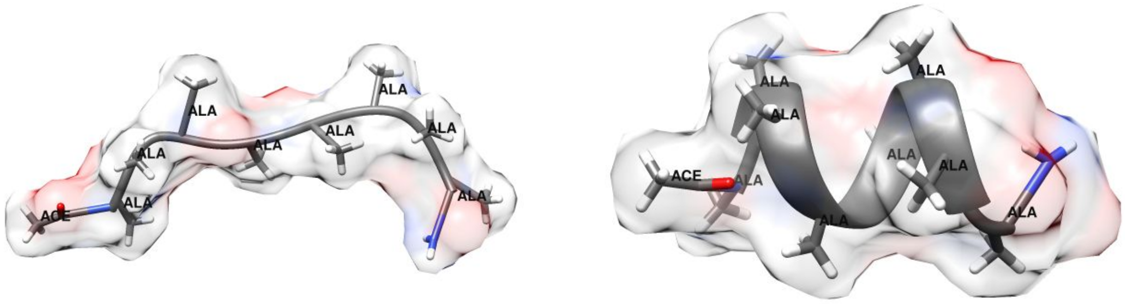

Figure S2. *The initial structures of the ALA_8 peptide used in simulations. Left: an extended structure ("e"), right an alpha-helical structure ("h").*

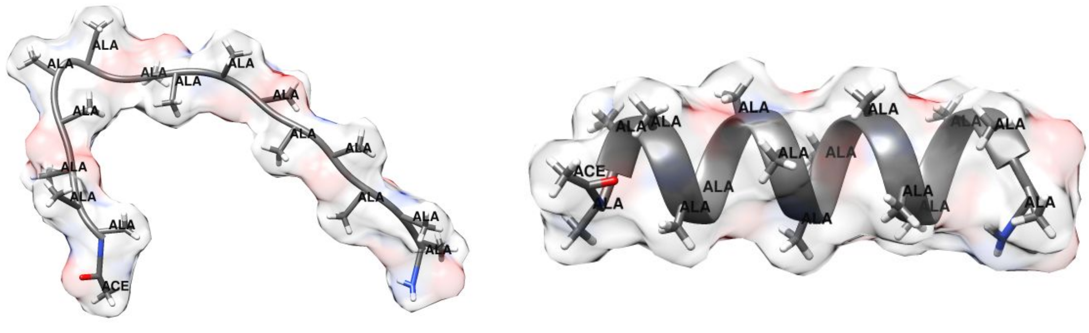

Figure S3. *The initial structures of the ALA_{15} peptide used in simulations. Left: an extended structure ("e"), right an alpha-helical structure ("h").*

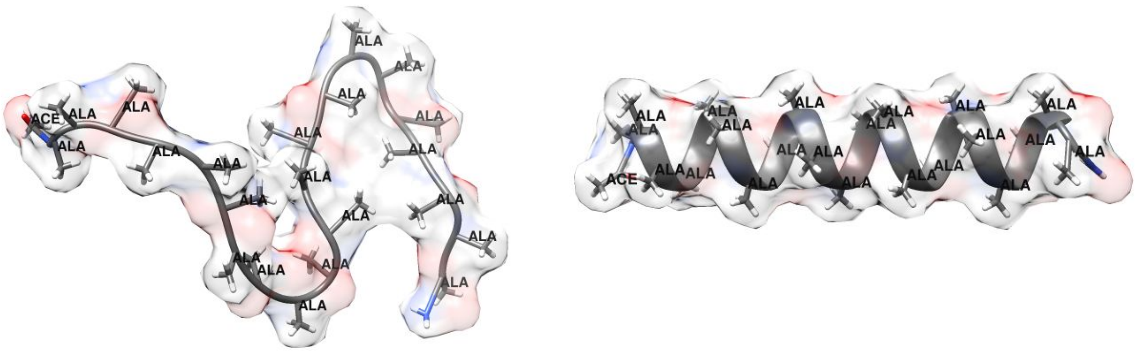

Figure S4. *The initial structures of the ALA_{21} peptide used in simulations. Left: an extended structure ("e"), right an alpha-helical structure ("h").*

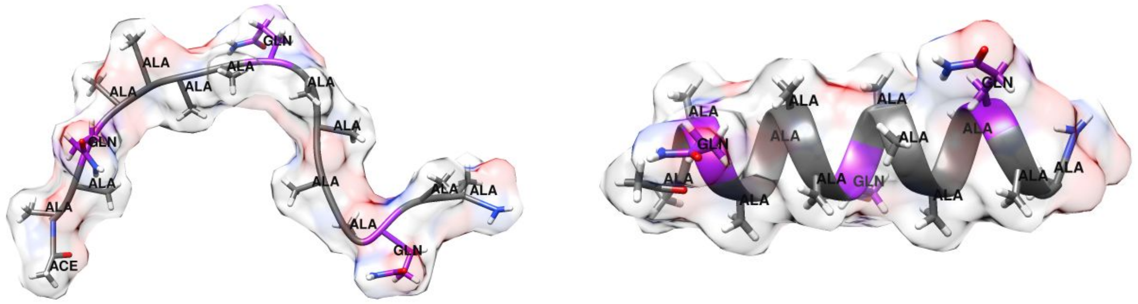

Figure S5. *The initial structures of the $(AAQAA)_3$ peptide used in simulations. Left: an extended structure ("e"), right an alpha-helical structure ("h").*

3) **Structural change in the course of MD: RMSD and hydrogen bond count.** Selected results for the studied peptides. The results for the ALA_n peptides have been published in the Supplementary Materials of the Ref. 1.

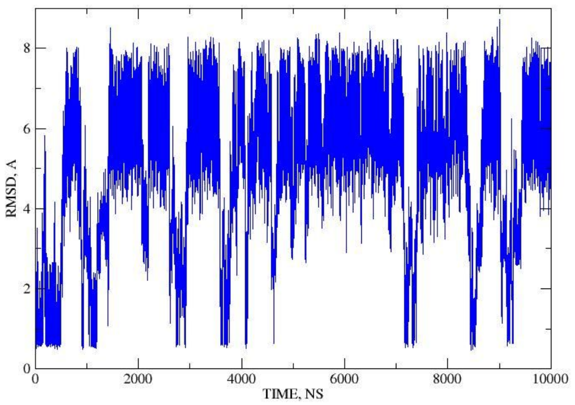

A. CA RMSD from helix: $(AAQAA)_3$ h

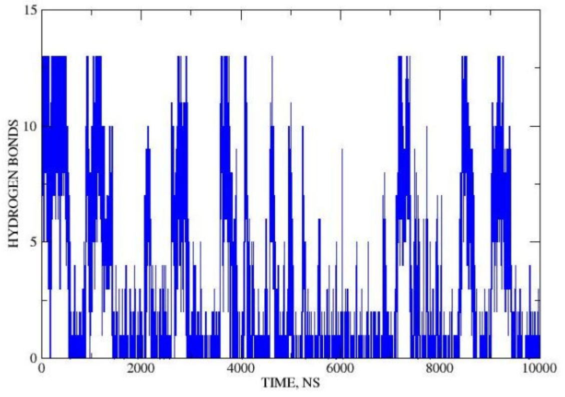

B. Hydrogen bond count: $(AAQAA)_3$ h

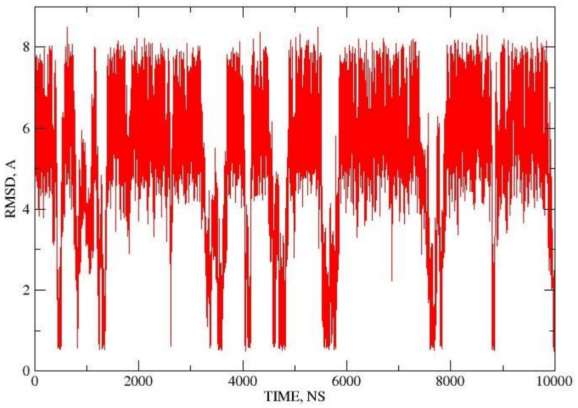

C. CA RMSD from helix: (AAQAA)$_3$ e

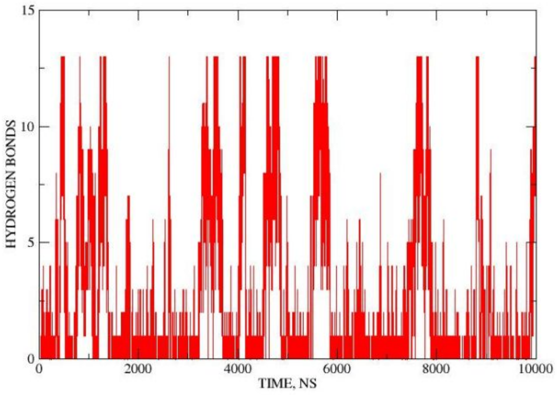

D. Hydrogen bond count: (AAQAA)$_3$ e

Figure S6. *RMSD and HB count for the (AAQAA)$_3$ peptide respectively for helical ("h") and extended ("e") conformations.*

4) Structural change in the course of MD: End-to-end distances for the KR1 peptide.

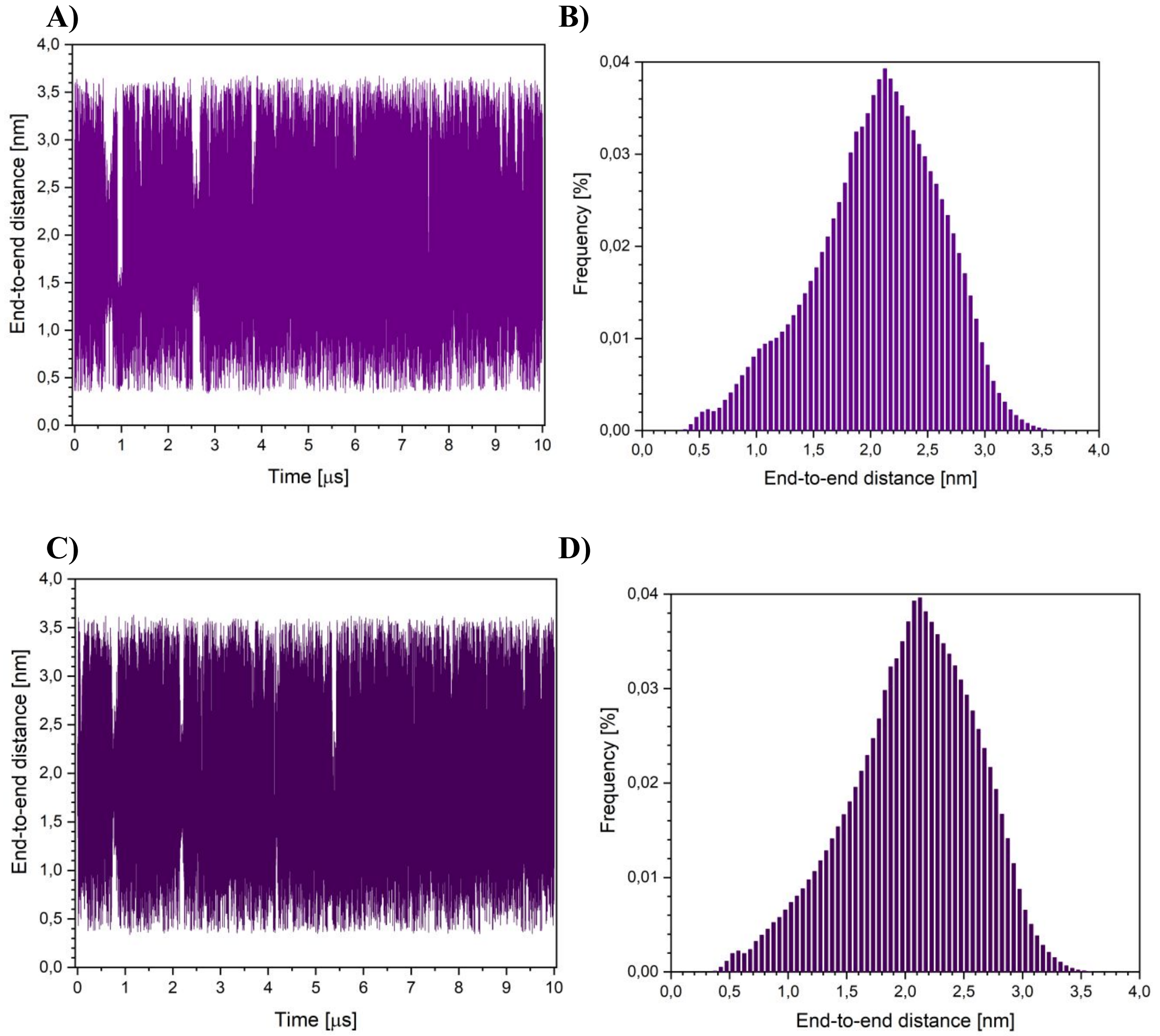

Figure S7. End-to-end distances for the probed trajectories for the KR1 peptide. *(A) End-to-end distances vs. time for trajectory starting from an extended peptide structure (trajectory*

„e”). (B) Histogram of (A). (C) End-to-end distances vs. time for trajectory starting from an alpha-helical structure (trajectory „h”). (D) Histogram of (C).

5) AlphaFold results for the KR1 peptide and their discussion.

AlphaFold AI simulations have been performed for the KR1 peptide (GN(AAQAA)$_2$G) to compare with the MD results and provide additional means to obtain the preferential conformations of the folded KR1. This algorithm has been recently made available to the public and its speed in obtaining good quality results have been well acclaimed.[2] Five separate trials ended with nicely folded structures, with a central part of the core and vicinity of the N-terminus of the peptide predicted to be helical at more than 90% lDDT score and with very similar folded structures over all trials, see **Fig. S8**. The resulted structures, all alpha-helical, see Fig. S10 (c-g) are very similar to an ideal alpha-helical structure used in our MD simulations, see Fig. S10(b). However, exact positions of terminal residues in the AlphaFold predictions are not well set for glycine at the N-terminal (residue “0” in Fig. S10(a)) as well as two alanines and one glycine at the C-terminal (residues “10-12” in Fig. S10(a)). This is reflected in lower values of the so-called IDDT score for these residues as compared with other residues, see Fig. S10(a). Such conclusions coincide, in general, with our MD simulations. However, detailed comparisons of AlphaFold results with MD simulations are usually taken with the grain of salt. This is because, AlphaFold predictions are based on machine learning for structures of much larger proteins, where the underlying force field has been smeared out to yield the observed result based on existing structures. Such existing structures have been stable due to „embedded” hydrophobic forces and evolutionary conserved long range interactions between residues far in sequence, but close in space. Such interactions are missing in the case of peptides.

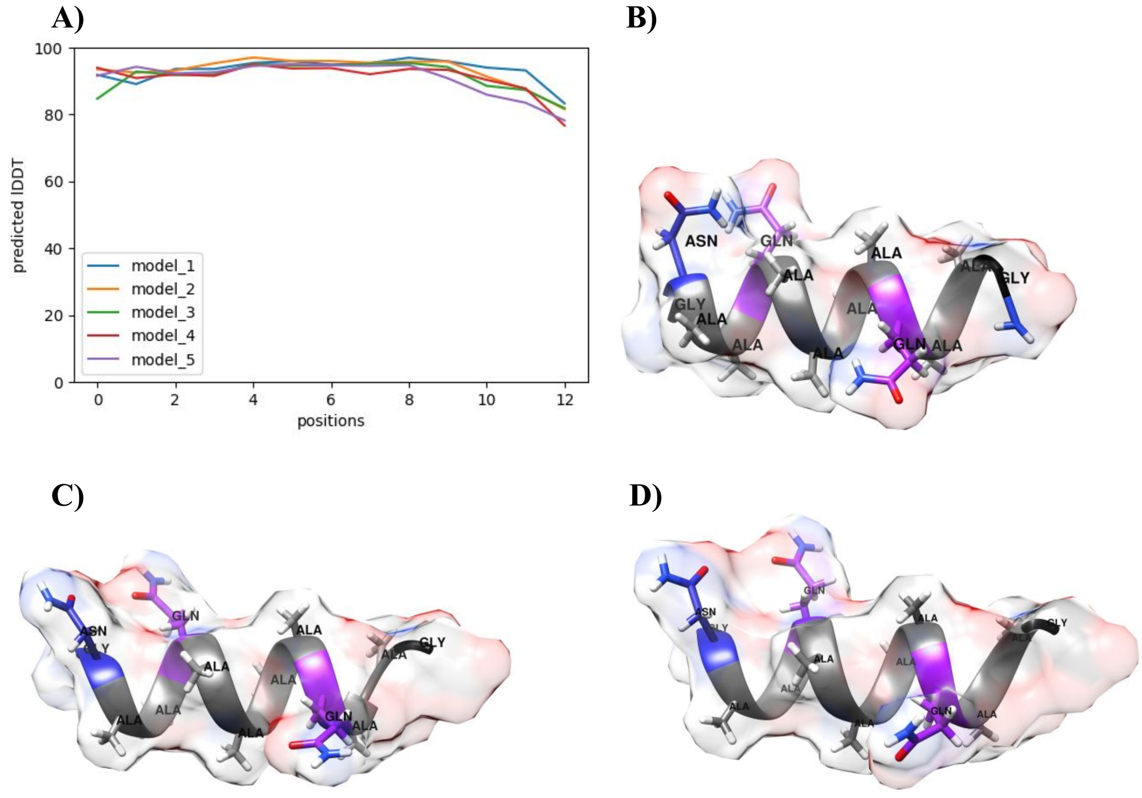

E)

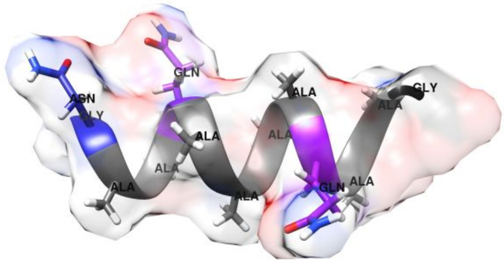

F)

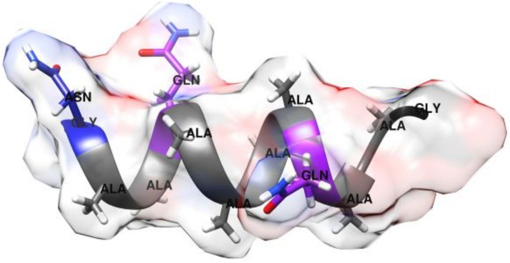

G)

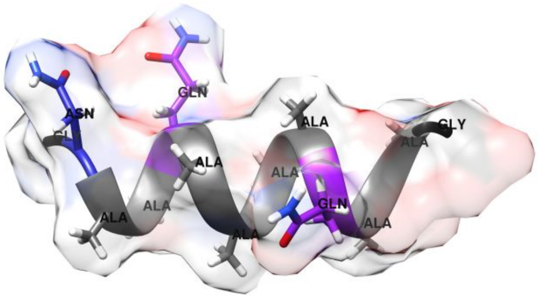

Figure S8. Folded structures of the KR1 peptide researched via an AlphaFold. *(A) Folding quality estimation over a sequence of the KR1 peptide, described by the lDDT score from the AlphaFold program in separate five trials. lDDT of 100% means perfectly helical arrangement. First residue was numbered "0", and the last 13th residue has a number "12". (B) Ideal alpha-helical structures used in our MD simulations. (C-G) Folded structures of the KR1 peptides obtained by AlphaFold algorithm in the studied cases.*

6) Values of the folding equilibrium constant (*K*) and corresponding free energy (Δ*G*) change upon folding obtained from the h+e HB counts for the studied peptides from helix fractions *p*:

$$K = \frac{p}{1-p} \quad and \quad \Delta G = -RT\, ln\frac{p}{1-p}$$

R the gas constant and T=300 K the temperature

System	*K*	Δ*G (kcal/mol)*
ALA_5 h+e	0.030 $\pm$ 0.004	2.09 $\pm$ 0.09
ALA_8 h+e	0.067 $\pm$ 0.016	1.59 $\pm$ 0.14
ALA_{15} h+e	0.332 $\pm$ 0.154	0.66 $\pm$ 0.28
ALA_{21} h+e	1.427 $\pm$ 0.530	-0.21 $\pm$ 0.22
$(AAQAA)_3$ h+e	0.277 $\pm$ 0.106	0.76 $\pm$ 0.23
KR1 h+e	0.055 $\pm$ 0.020	1.73 $\pm$ 0.22

Table S2. *Values of the equilibrium constant (K) obtained from the averaged (h+e) HB counts for the studied peptides.*

7) **Fits to normalized RMSD autocorrelation function to obtain the correlation times for the KR1 peptide.**

We have performed single and exponential fits to the normalized autocorrelation functions obtained for both analyzed trajectories ("e" and "h") from the RMSD data. The quality of the fits was assessed via a reduced χ^2 coefficient, which takes into account a larger number of parameters for double exponential fits than for a single exponent. Noteworthy, reduced χ^2 for a good fit it should be much less than unity. Table 1 presents the obtained results, which show clearly that double exponentials fit the data much better than single exponentials and that an allowance for a small offset provides a substantial improvement in the double exponential fitting. Therefore, the results obtained in the case of double exponential fits with an offset will be used later.

Reduced χ^2 ($x10^{-4}$)	Trajectory "e" **see Fig. S11**	Trajectory "h" **see Fig. S12**
Single exponential fit	5,79	9,11
Single exponential fit with an offset	5,77	8,92
Double exponential fit	2,15	3,50
Double exponential fit with an offset	1,62	0,44

Table S3. *Values of the reduced χ^2 obtained for fitting exponential decay to the normalized autocorrelation function for the investigated trajectories for the KR1 peptide. Best results have been obtained for a double exponential fit with an offset.*

Correlation times (ns)	Trajectory "e"	Trajectory "h"
τ_{c1}	3,93 ± 0,02	1,69 ± 0,01
$\boldsymbol{\tau_{c1, ave}}$	**2,81 ± 1,12**	
τ_{c2}	77,01 ± 0,20	39,36 ± 0,13
$\boldsymbol{\tau_{c2, ave}}$	**58,18 ± 18,83**	

Table S4. *Correlation times obtained in the case of double exponential fits with allowance for a small offset, see Table S3. Fitting errors are much smaller than respective maximum deviations between averaged values. Thus, maximum deviations are used instead.*

The graphs showing actual fits discussed above are presented below in **Figs. S9-S10**.

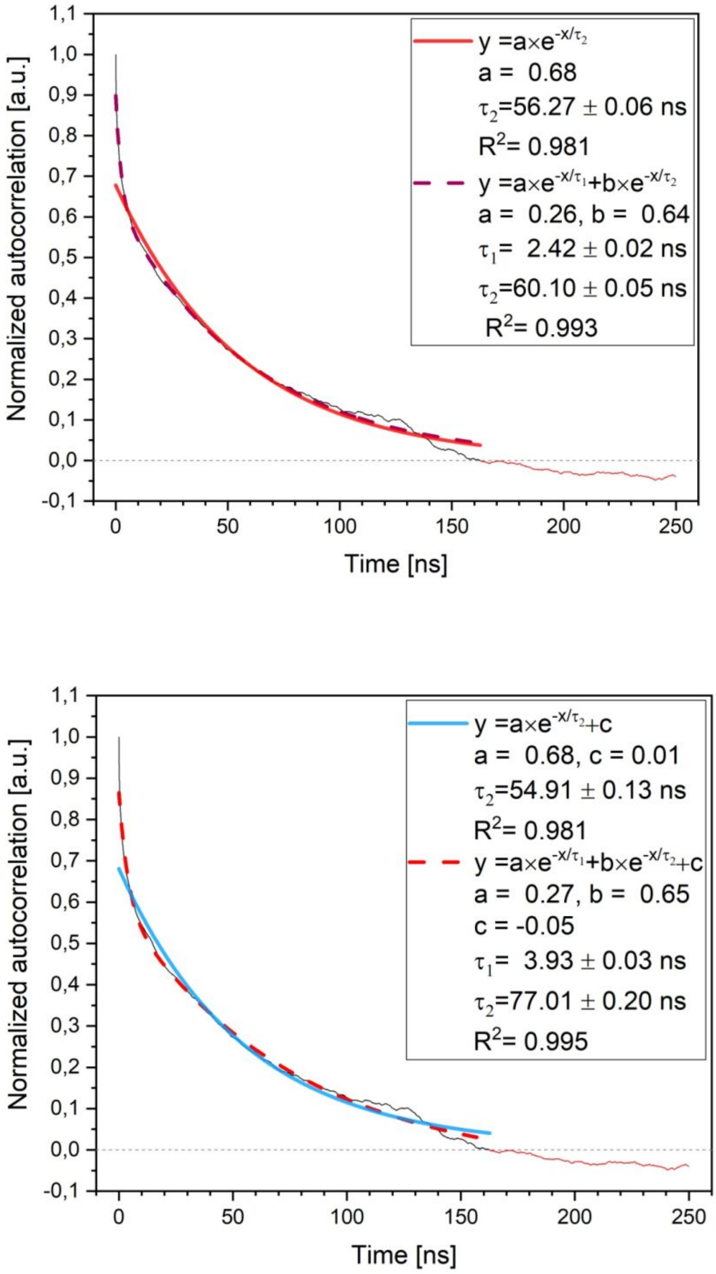

Figure S9. Fits to the normalized autocorrelation function for the trajectory „e" for the KR1 peptide. *Top: without any offset; bottom: with an offset.*

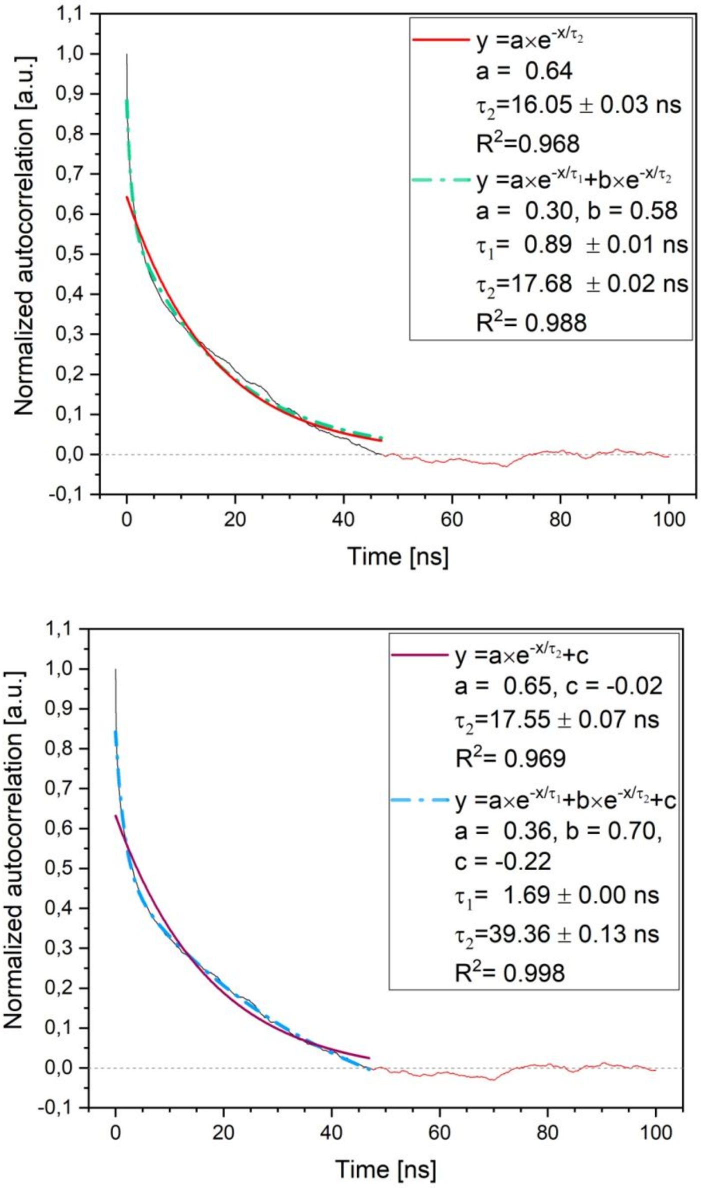

Figure S10. Fits to the normalized autocorrelation function for the trajectory „h" for the KR1 peptide.

8) Obtaining the correlation times for other studied peptides: $(AAQAA)_3$ and ALA_n peptides.

Similar analysis as described above has been applied to the $(AAQAA)_3$ peptide, and the results of the relaxation times obtained from a 2-exponential fits to the RMSD ACF (auto-correlation function) with small offset allowance are:

h trajectory: fast time $\tau_1 = 0.82 \pm 0.01\ ns$ and slow time $\tau_2 = 108 \pm 15.9\ ns$

e trajectory: fast time $\tau_1 = 0.72 \pm 0.01\ ns$ and slow time $\tau_2 = 85.9 \pm 11.3\ ns$

average: fast time $\tau_1 = 0.77 \pm 0.01\ ns$ and slow time $\tau_2 = 97.0 \pm 9.8\ ns$

Due to the difference obtained between "e" and "h" trajectories being larger than individual errors arising from fitting, the maximum scatter between trajectories will be used as corresponding error for average values of the correlation times to yield average values with errors: Overall, we get: fast time $\tau_1 = 0.77 \pm 0.05\, ns$ and slow time $\tau_2 = 97.0 \pm 11.0\, ns$.

Results for the ALA_n peptides are gathered in the Table S5 below.

	h		e		mean	
	tau1	tau2	tau1	tau2	tau1	tau2
ALA5	0,2	2,2	0,1	1,8	0,15	2
ALA8	0,95	12	0,7	13	0,825	12,5
ALA15	1,8	100	1,8	77	1,8	88,5
ALA21	6,8	170	7	200	6,9	185

Table S5. *Correlation times in the case of $(ALA)_n$ peptides obtained from the MD ACF RMSD fits.*

9) **The data used to plot Figure 6 in the main paper.** First column is the number of hydrogen bonds. Second column is the average end-to-end distance for a given number of hydrogen bond. Third column is an error of the second column.

$(AAQAA)_3$ peptide

```
 0   2.0980   0.0173
 1   2.0542   0.0302
 2   2.0907   0.0380
 3   1.9830   0.0500
 4   1.9234   0.0677
 5   1.8879   0.0702
 6   2.0148   0.0483
 7   2.0294   0.0511
 8   1.9779   0.0660
 9   1.9855   0.0819
10   2.1178   0.0418
11   2.1573   0.0144
12   2.1196   0.0006
13   2.1081   0.0005
```

KR1 peptide

```
 0   2.0942   0.0189
 1   1.9805   0.0341
 2   1.9861   0.0363
 3   1.8968   0.0596
 4   1.7632   0.0973
 5   1.7846   0.0959
 6   1.9066   0.0490
 7   1.8875   0.0402
```

8	1.8529	0.0350
9	1.8158	0.0206
10	1.8515	0.0017

ALA5

0	1.1482	0.0035
1	0.7298	0.0068
2	0.6283	0.0017
3	0.6235	0.0004

ALA8

0	1.7047	0.0064
1	1.2960	0.0163
2	1.2297	0.0196
3	1.2484	0.0160
4	1.1698	0.0117
5	1.0699	0.0005
6	1.0569	0.0005

ALA15

0	2.1261	0.0160
1	2.0540	0.0345
2	2.1019	0.0333
3	2.0161	0.0431
4	1.8554	0.0795
5	1.8995	0.0672
6	1.9926	0.0665
7	1.9482	0.0638
8	1.9475	0.0626
9	2.0087	0.0567
10	2.1220	0.0272
11	2.1306	0.0138
12	2.1173	0.0006
13	2.1047	0.0004

ALA21

0	2.2069	0.0364
1	2.1191	0.0755
2	2.2127	0.0575
3	2.1688	0.0572
4	2.0763	0.0697
5	2.1642	0.0535
6	2.1863	0.0507
7	2.0955	0.0661
8	2.0843	0.0712
9	2.1995	0.0522
10	2.2631	0.0664
11	2.2405	0.0748
12	2.3112	0.1010
13	2.3949	0.0966
14	2.5366	0.0472

15	2.6229	0.0187
16	2.6729	0.0090
17	2.7163	0.0052
18	2.7369	0.0040
19	2.7351	0.0056

References

(1) Kuczera, K.; Szoszkiewicz, R.; He, J.; Jas, G. S. Length Dependent Folding Kinetics of Alanine-Based Helical Peptides from Optimal Dimensionality Reduction. *Life* **2021**, *11* (5), 1–16. https://doi.org/10.3390/life11050385

(2) Jumper, J., Evans, R., Pritzel, A. *et al.* Highly accurate protein structure prediction with AlphaFold. *Nature* **596,** 583–589 (2021). https://doi.org/10.1038/s41586-021-03819-2
